# N-Acetyldopamine Dimer Attenuates DSS-Induced Ulcerative Colitis by Suppressing NF-κB and MAPK Pathways

**DOI:** 10.3389/fphar.2022.842730

**Published:** 2022-04-06

**Authors:** Li-Jun Huang, Yu-Mei Wang, Lei-Qiang Gong, Chao Hu, Yu Gui, Chen Zhang, Xue Tan, Xian-Kuo Yu, Yi-Le Liao, Yan Luo, Yu-Qin Tang, Yi-Fei Dai, Yun Deng, Dong Wang, Da-le Guo

**Affiliations:** ^1^ State Key Laboratory of Southwestern Chinese Medicine Resources, School of Basic Medical Sciences, Chengdu University of Traditional Chinese Medicine, Chengdu, China; ^2^ School of Pharmacy, Chengdu University of Traditional Chinese Medicine, Chengdu, China; ^3^ Department of Basic Medical Sciences, School of Medicine, Tsinghua University, Beijing, China

**Keywords:** N-acetyldopamine dimer, ulcerative colitis, inflammation, NF-κB pathway, MAPK pathway

## Abstract

Ulcerative Colitis (UC) is a major form of chronic inflammatory bowel disease of the colonic mucosa and exhibits progressive morbidity. There is still a substantial need of small molecules with greater efficacy and safety for UC treatment. Here, we report a N-acetyldopamine dimer (NADD) elucidated (2R,3S)-2-(3′,4′-dihydroxyphenyl)-3-acetylamino-7-(N-acetyl-2″-aminoethyl)-1,4-benzodioxane, which is derived from traditional Chinese medicine *Isaria cicadae*, exhibits significant therapeutic efficacy against dextran sulfate sodium (DSS)-induced UC. Functionally, NADD treatment effectively relieves UC symptoms, including weight loss, colon length shortening, colonic tissue damage and expression of pro-inflammatory factors in pre-clinical models. Mechanistically, NADD treatment significantly inhibits the expression of genes in inflammation related NF-κB and MAPK signaling pathways by transcriptome analysis and western blot, which indicates that NADD inhibits the inflammation in UC might through these two pathways. Overall, this study identifies an effective small molecule for UC therapy.

## Introduction

Ulcerative Colitis (UC) is a chronic inflammatory bowel disease that shows increasing incidence worldwide (Ordás et al., 2012; Ng et al., 2017). The incidence of UC in North America, Europe, and Oceania is greater than 15 per 100,000 person-years (Du and Ha, 2020). The annual incidence of UC, among Asian countries, is relatively high in China with a mean of 1.18 per 100,000 person-years (Zhou et al., 2021). The occurrence of UC is influenced by many factors, including genetic, environmental, and luminal factors (Ordás et al., 2012). The pathophysiology of UC is multifaceted and its etiology is still not completely understood.

Mucosal inflammation in UC usually starts from the rectum and gradually extends to part of or the entire colon (Ordás et al., 2012). Intestinal barrier disruption caused by an inflammatory cascade may lead to UC chronicity. Medical treatment of UC mainly focuses on reducing inflammation and uses mesalazine, corticosteroids, immune-suppressive drugs, and TNF-α monoclonal antibodies (Ordás et al., 2012). Nevertheless, side effect, safety, and tolerance issues with these drugs indicate an urgent need for effective agents with fewer unwanted properties. Natural products from traditional Chinese medicine might be promising resources of such molecules for UC treatment.

**FIGURE 1:**
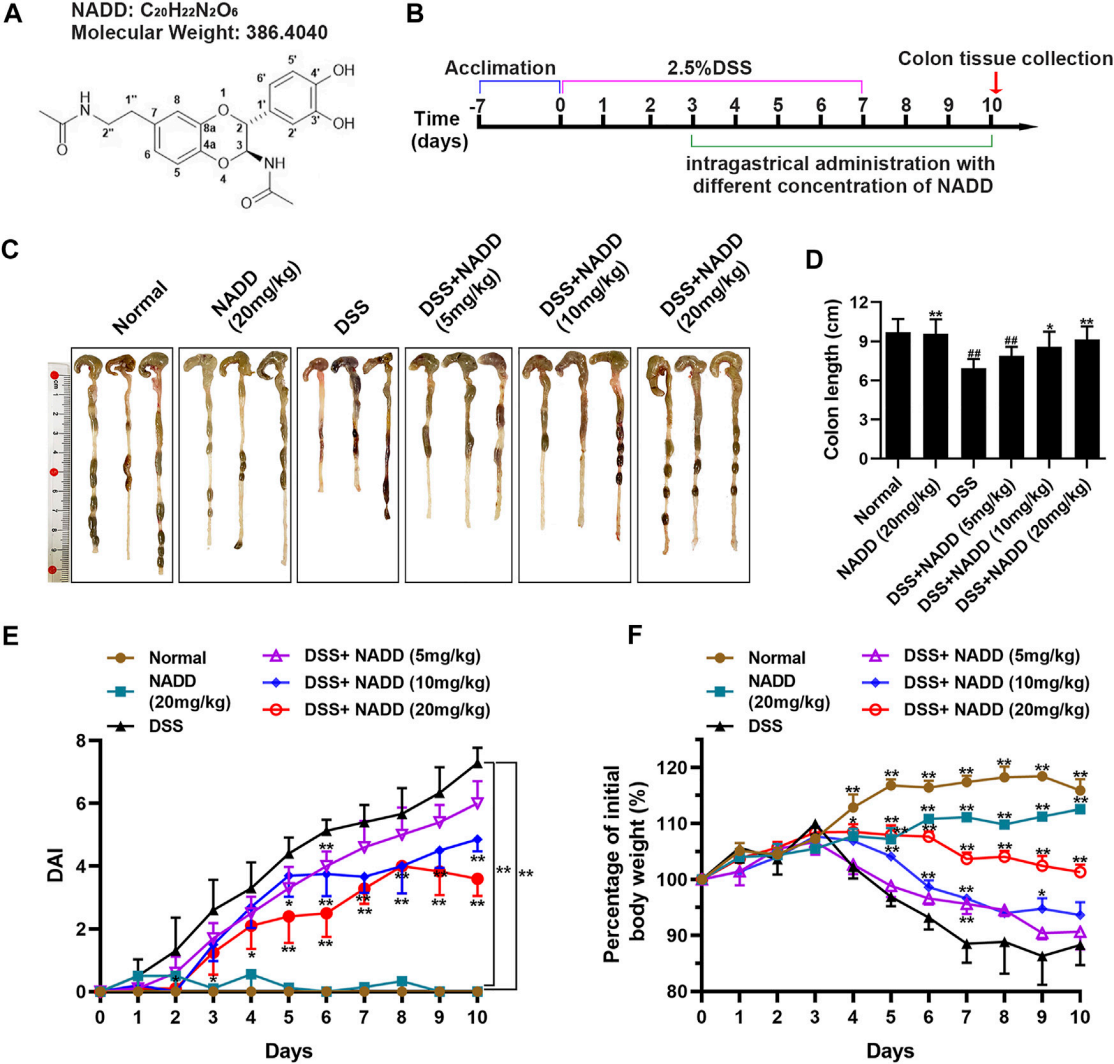
The therapeutic effect of NADD on UC. **(A)** Molecular structures of NADD **(B)** Experiment flow diagram. **(C)** Images of murine colons among different groups of mice **(D)** Histogram of colon length after treatment with different concentrations of NADD. **(E)** DAI of mice among different treatment groups. **(F)** Body weight changes of mice among different treatment groups. Differences were statistically significant when compared with mice in the DSS group (^*^ *p* < 0.05, ^**^ *p* < 0.01).

*Isaria cicadae* is a homologous medicine and food that displays anti-oxidation, anti-aging, anti-inflammation, and antitumor properties, ameliorates renal dysfunction, and shows remarkable immune modulation (Hua et al., 2016; Xu et al., 2018). *I. cicadae* has been used to cure inflammatory bowel disease in traditional Chinese medicine. The Compendium of Materia Medica, for instance, indicates that *I. cicadae* can cure malaria. A phytochemical search for active constituents in *I. cicadae* led to the isolation an N-acetyl dopamine dimer (NADD, Figure 1A), which was elucidated as (2R,3S)-2-(3′,4′-dihydroxyphenyl)-3-acetylamino-7-(N-acetyl-2″-aminoethyl)-1,4-benzodioxane. It was reported this compound exhibits antioxidant and anti-inflammatory properties (Xu et al., 2006), but its efficacy for the treatment of UC and underlying mechanism of anti-inflammatory activity are unclear.

Dextran sulfate sodium (DSS) induces changes in the gut microbiome, especially causing an increase in the number of gram-negative anaerobes (Okayasu et al., 1990). Mice treated with DSS mimic severe pathophysiological damage seen in human UC (Okayasu et al., 1990; Cooper et al., 1993; Ohkawara et al., 2002; Forbes et al., 2004). Clinical manifestations of UC induced by DSS include weight loss, diarrhea, and occult blood in stools. These issues are summarized with the DAI (Perse and Cerar, 2012). Hence, DSS-induced UC mice are considered a good model to study the effects and potential mechanisms of pharmacologic agents in treating inflammatory colon disease (Cooper et al., 1993).

This study evaluated the efficacy of NADD toward UC and explored underlying anti-inflammatory mechanisms. DSS-induced UC mouse model was used to evaluate protective effects of NADD. Endpoints for assessing disease activity index (DAI), colon length, histopathological changes, as well as the secretion and gene expression of cytokines. Subsequently, lipopolysaccharide (LPS)-induced RAW264.7 macrophages were employed to investigate anti-inflammatory mechanisms. Transcriptional analysis combined with western blotting of LPS-induced RAW264.7 macrophages before and after NADD treatment was used to reveal related signaling pathways.

## Materials and Methods

### Materials

*I. cicadae* was collected from a suburb of Ya’an, Sichuan province, China. DSS (40 KD) was purchased from Seebio Biotechnology Co., Ltd (Shanghai, China). TLR4 protein (purity ≥ 87%) was purchased from Sino biological. Bovine serum albumin (BSA) was purchased from Gemini. Animal Total RNA Isolation and Cell Total RNA Isolation Kits (RE-03014 and RE-03014113, respectively), RT EasyTM II (Master Premix for first-strand cDNA synthesis for Real-Time PCR RT-01022), and Real-Time PCR EasyTM-SYBR Green I (QP-01012) were purchased from Foregene (Chengdu, China). RNA Isolater Total RNA Extraction Reagent was purchased from Vazyme (R401-01, Nanjing, China). The Immobilon Western Chemiluminescent HRP Substrate was from Millipore (WBKLS0500, United States).

### Preparation and Characterization of the NADD

Air-dried powder of *I. cicadae* (10 Kg) was extracted using 95% aqueous MeOH (3 × 100 l) under reflux. A crude extract was obtained after removing the solvent under reduced pressure. This extract was then suspended in distilled water (10 l) and placed on a macro-porous resin D101 column (D × L: 20 × 120 cm). The column was eluted with MeOH-H_2_O (0:100, 30:70, 60:40, 100:0, V/V) to yield four fractions (A-D). Fraction C (150 g) was placed on a silica gel column (100–200 mesh, D × L: 15 × 80 cm) and eluted with a PE-acetone gradient (100:0→1:1, V/V) to afford eight subfractions (C1-C8). Fractions C2 (4.1 g), C3 (1.9 g), and C4 (3.9 g) were combined, guided by TLC, and passed through a Sephadex LH-20 column (D × L: 2 × 180 cm, CHCl_3_-MeOH, 1:1). Finally, the combined fractions were further purified by preparative HPLC (5 μm, 120 A, D × L: 10 × 250 mm) with MeOH-H_2_O (30:70, 10 ml/min) to yield NADD (1.26 g, purity ≥98%, (Supplementary Figures S1, S2).

### Animals

All animal experiments were conducted as per protocols accredited by the Animal Welfare Committee of Chengdu University of Traditional Chinese Medicine, and in accordance with the National Institutes of Health Guide for the Care and Use of Laboratory Animals.

Imprinting control region male mice (7 weeks old, 30–35 g) were supplied by Specific Pathogen Free (SPF) Biotechnology Co., Ltd (Beijing, China) and housed in the animal facility of the Laboratory Animal Center of Chengdu University of Traditional Chinese Medicine, under standard conditions. All mice were provided food and distilled water. Animal experiments were designed following reported procedures (Ma et al., 2018; Shi et al., 2020; Wang et al., 2021; Zhang et al., 2021). After one week of acclimatization, mice were randomly divided into six groups (*n* = 8 per group): normal group (normal), NADD administration group (NADD (20 mg/kg)), UC model group (DSS), rescue UC model groups administrated different concentrations of NADD—DSS + NADD (5 mg/kg), DSS + NADD (10 mg/kg), DSS + NADD (20 mg/kg). NADD was dissolved in normal saline.

Mice administered DSS or DSS combined with NADD were supplied with freely accessible 2.5% DSS in drinking water for first 7 days. DSS was discontinued in favor of distilled water for the last 3 days (days 8–10). Normal mice and mice administered NADD (20 mg/kg) were provided with only distilled water for all 10 days. From the third day, mice in DSS + NADD (5 mg/kg), DSS + NADD (10 mg/kg), DSS + NADD (20 mg/kg) groups were administrated low, middle, and high concentrations of NADD by gavage. Mice in normal and DSS groups were administrated the same volume of normal saline by gavage as parallel controls. Body weight and DAI were recorded daily during the experiment. DAI was evaluated on the last day as the sum of body weight loss, diarrhea, and fecal bleeding (Wang et al., 2021). All groups of mice were sacrificed and colon tissues were obtained; colon length of each mouse was measured. Colon tissues were divided into five parts for different analyses.

### Morphological Analysis

Colon tissues were fixed with 4% paraformaldehyde for 24 h and dehydrated with 30% sucrose at 4°C for 72 h. Tissues were then embedded in paraffin. Embedded tissues were cut into 5 μm- thick sections and stained with hematoxylin-eosin (HE) or Periodic Acid-Schiff (PAS) (Naeem et al., 2020). Stained sections were visualized and images were captured by light microscopy.

### RT-PCR of Colon Tissues

Total RNA of colon tissues was isolated using an Animal Total RNA Isolation Kit, and mRNA was reverse transcribed into cDNA using an RT EasyTM II Kit following the manufacture’s protocol. Expression of target genes was assessed with RT-PCR using Analytikjena (qTOWER3 G, Germany) and Real-Time PCR EasyTM-SYBR Green I. Sequences of primers TNF-α, IL-1β, iNOS, IL-6 and β-actin were previously reported (Wang et al., 2014). The primer sequences of Ikbkb, CCL2, Rela (p65), Nfkb1 (p50) and Nfkb2 (p52) were designed as previously described (Listwak et al., 2013).

### ELISA Analysis of Colon Tissues

Colon tissues were lysed in PBS using a tissuelyzer (Servicebio, KZ-III F, China), then centrifuged and supernatants collected. TNF-α, IL-1β and IL-6 in supernatants were assessed using enzyme-linked immunosorbent assay (ELISA) kits (Multi Science, China), following the manufactures instruction. Absorbance was measured at 450 nm.

### Cell Culture

Murine leukemic monocyte macrophage cells (RAW264.7 macrophages) were cultured in a DMEM complete medium with 10% fetal bovine serum and 1% penicillin-streptomycin at 37°C in a humidified atmosphere with 5% CO_2._ In cell culture experiments, both NADD and LPS were dissolved in DMSO, therefore, DMSO was used as control.

### Cell Viability Assay

RAW 264.7 macrophages were seeded into 96-well plates at a density of 8 × 10^3^ cells/well. Cells achieved a density of 60–70% by the next day. Cells were then pre-treated with different concentrations of NADD (0 μM, 10 μM, 30 μM, 60 μM, 100 μM, 200 μM) for 1 h, followed by co-treating with or without 1 ug/mL LPS for 24 h. Subsequently, 10 μl of Cell Counting Kit-8 reagent (CCK8, MCE, HY-K0301) was added to each well and incubation continued for 3 h. Finally, optical density of formazan was measured at 450 nm using a microplate reader to assess cell viability (Thermo, Varioskan, MA, United States).

### Transcriptome Sequencing (RNA-Seq)

RAW264.7 macrophages were seeded into 10 cm plates. When cells achieved a density of 60–70%, they were pre-treated with NADD at the concentration of 60 μM or without NADD for 1 h, followed by co-treatment with or without 1 μg/ml LPS for 24 h and generated three groups (DMSO group, LPS group, NADD + LPS group). A total of 5 × 10^6^ cells were collected and lysed with RNA Isolater Total RNA Extraction Reagent. Lysates or fresh colon tissues were sent for transcriptome sequencing at Annoroad Biotechnology Co., Ltd (Zhejiang, China): Purity, concentration, and integrity of total RNA samples were assessed before further analysis. A cDNA library was obtained after amplification and purification. Finally, the cDNA library was sequenced using the Illumina NovaSeq 6000 platform with a 150-bp pair-end sequencing strategy to generate raw reads. Clean reads were obtained after removing adapter, poly-N sequences, and inferior quality reads. These reads were mapped to the *Mus musculus* (GRCm39 (Howe et al., 2021)) reference genome sequence using HISAT2 (Kim D. et al., 2019) tools. We assembled transcripts using HTSeq (Anders et al., 2015) for each sample to obtain gene read counts. Differential gene expression was assessed using DESeq2. Only genes with *p-*value < 0.05 and fold change value ≥ 1.5 were considered as significantly differentially expressed. We use cluster profiler (Wu et al., 2021) to assess KEGG pathway enrichment of DEGs. Volcano and heatmap plots were drawn in R. We used the upregulated genes and downregulated genes after LPS treatment as the background gene sets datasets, respectively. The upregulated and downregulated genes after LPS + NADD treatment were sorted according to the fold changes and used as input data for GSEA analysis. The related results showed whether the upregulated and downregulated genes were enriched in the term of background gene sets.

### Western Blotting

RAW264.7 macrophages were seeded into 6-well plates at a density of 5 × 10^5^ cells/well. When the cells achieved a density of 60–70%, they were pre-treated with different concentrations of NADD (10 μM, 30 μM, 60 μM) for 1 h, followed by co-treatment with or without 1 ug/mL LPS for 24 h. Subsequently, cells were collected and lysed with RIPA lysis buffer at 4°C for 30 min, centrifuged, and supernatants collected. The protein concentrations were then analyzed using a BCA protein assay kit (Beyotime, P0012, China) following the manufacturer’s instructions. Aliquots with equal amounts of protein were loaded into wells of SDS-PAGE gels, subjected to electrophoresis and protein transferred to PVDF membranes (Ju et al., 2021). After blocking with 10% BSA, membranes were incubated with primary antibodies at 4°C overnight. Primary antibodies were: TLR4 (1:4000, Proteintech, 66350-1-Ig, China), IKK-α (1:1,000, CST, 11930 s, United States), p-IKK-α (1:1,000, CST, 2,697 s, United States), NF-κB-P65 (1:1,000, CST, 8,242 s, United States), p-NF-ΚB-P65 (1:1,000, CST, 3,033 s, United States), IκB-α (1:1,000, CST, 4812 s, United States), p-IκB-α (1:1,000, CST, 5,209 s, United States), P38 (1:1,000, CST, 8,690 s, UnitedStates), p-P38 (1:1,000, CST, 4511s, United States), R-iNOS (1:1,000, NOVCCS, NB300-605SS, United States), COX-2 (1:1,000, abcam, ab179800, United States), α-tubulin (1:20,000, proteintech, 66031-1-Ig, China). Membranes were washed three times, and incubated with species-specific secondary antibodies at room temperature for 2 h: HRP-Anti-mouse-IgG (1:5,000, servicebio, GB23301, China), and HRP-Anti-rabbit-IgG (1:5,000, servicebio, GB23303, China). Finally, targeted proteins were visualized using HRP substrate for immunoblotting and detected using a Chemiluminescent Imaging System (MiniChemi 610, China). The immunoblots of targeted proteins were analyzed using ImageJ.

### Statistical Analysis

All inter-group comparisons were assessed using one-way analysis of variance by SPSS 26 and reported as mean ± standard deviation (SD). *p* < 0.05 was considered statistically significant, and a post-hoc analysis was done to determine which comparisons were significant.

## Results

### NADD Alleviates Colitis Symptoms

The UC model was specifically constructed by receiving 2.5% DSS in drinking water on days 0–7, followed by normal drinking water for the last 3 days before sacrifice. Subsequently, different concentrations of NADD were administered orally from the fourth day to assess their impact on colitis. Parallel control groups (normal and DSS) were administrated with normal saline (Figure 1B). The UC model was constructed successfully as indicated by findings in normal and DSS group mice (Figures 1C–E). Further, mice administered NADD were compared to UC model mice to assess the therapeutic effect of NADD. The colon in DSS group mice appeared red, bleeding and shortened. In contrast, the colon for animals administered NADD gradually improved and became similar to normal mice. No differences were observed between colons from normal and NADD treated mice. Colon length shortened significantly in mice exposed to DSS when compared with normal animals (Figure 1C, ^##^ *p* < 0.01). However, NADD administration gradually reversed this shortening increasing oral dose (Figure 1D, ^*^ *p* < 0.05, ^**^ *p* < 0.01). No changes in DAI were noted between colons from normal and NADD treated mice. Compared with normal and NADD treated mice, all the DAI increased with time in DSS and DSS + NADD treated animals. However, the increase of DAI in DSS group was most notable over time, because NADD administration reversed this increase of DAI induced by DSS in a dose-dependent manner (Figure 1E, ^*^ *p* < 0.05, ^**^ *p* < 0.01). The body weight of DSS-exposed mice decreased over time, while the body weight of normal and NADD treated animals increased gradually. Compared with DSS group mice, the body weight of DSS + NADD treated animals increased significantly each day: In the low concentration group [DSS + NADD (5 mg/kg)], there were a trend of body weight increasing on days 5–10, but only on day 7, the increase was significantly; In the middle concentration group [DSS + NADD (10 mg/kg)], the trends of body weight increasing were more notable on days 4–10, and there were significantly increasing of body weight on days 5, 6, 7, and 9; In the highest concentration group [DSS + NADD (20 mg/kg)], the trends of body weight increasing were most notable, and there were significantly increasing of body weight on days 4, 5, 6, 7, 8, 9, and 10. To sum up, increases were most significant for mice administered NADD+20 (mg/kg) (Figure 1F, ^*^ *p* < 0.05, ^**^ *p* < 0.01). NADD thus relieved the symptoms of DSS-induced UC in a dose-dependent manner.

### NADD Reduced Histologic Injury of Colitis Induced by DSS

**FIGURE 2:**
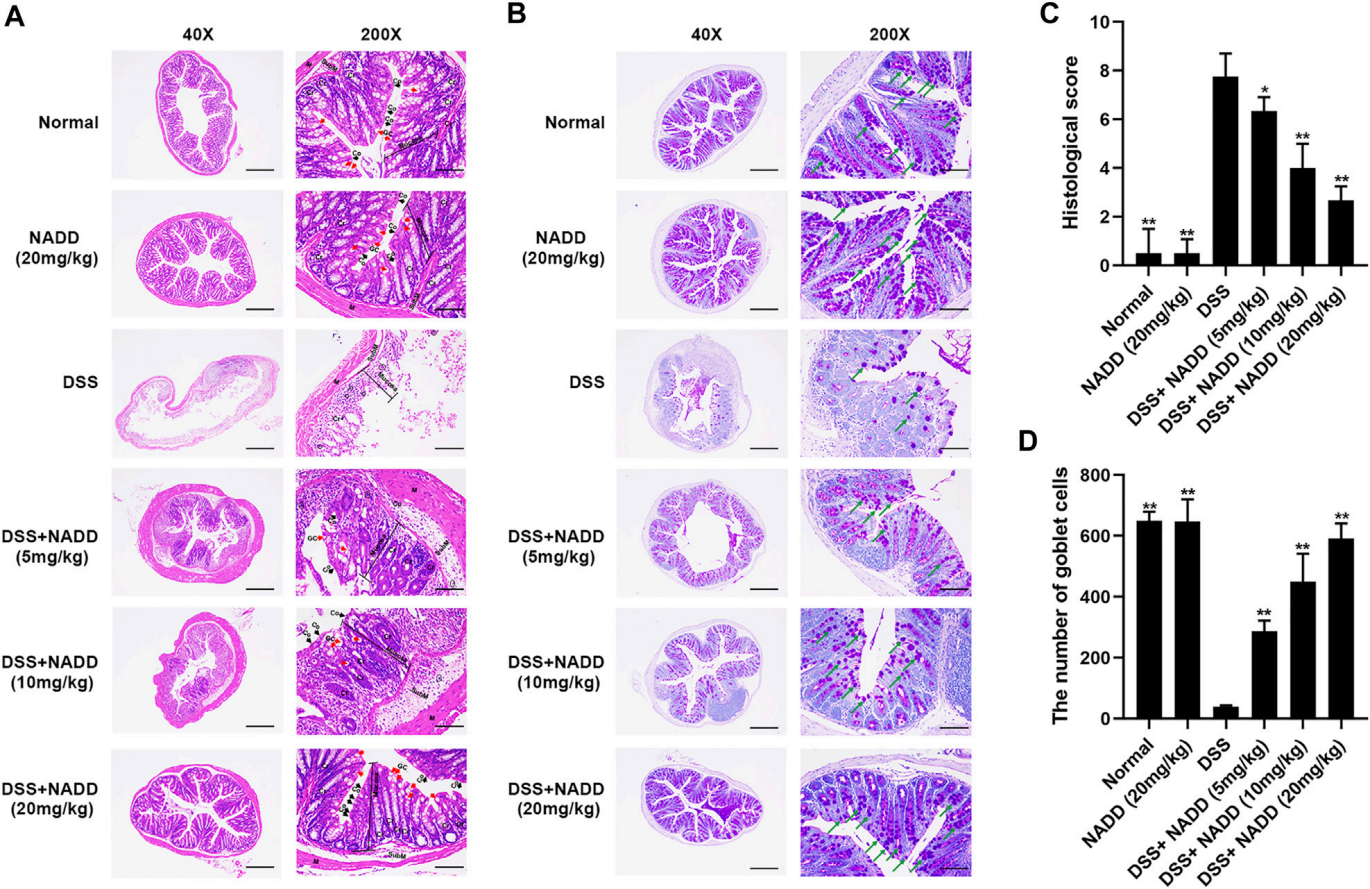
HE and PAS staining. **(A)** Representative images of HE staining sections of colon; GC, goblet cells (red arrows); Cr, Crypts; Co, Columnar colonocytes (black arrows); SubM: Submucosa; Cells in the circle are Neutrophils. **(B)** Representative images of PAS staining; Amaranth granules are mucin glycoproteins (green arrows). **(C)** Histological scores of colon tissues with or without DSS induction and with or without administration of NADD. **(D)** The number of goblet cells of colon tissues with or without DSS induction and with or without administration of NADD. Differences were statistically significant when compared with the DSS group mice (^*^ *p* < 0.05, ^**^ *p* < 0.01).

Histological damage in colon tissues from DSS-induced UC reflected injury seen in human UC. Pathology included the disappearance of colonocytes, mucin depletion, decreases in goblet cells and crypts, neutrophil infiltration of lamina propria, and submucosa, and edematous submucosa (Perse and Cerar, 2012). Normal mucosa, thin submucosa and muscularis was found in the colon of normal and NADD (20 mg/kg) treated mice, and columnar colonocytes, goblet cells and long crypts were found in the mucosa (Figure 2A). The mucosa layer in DSS-exposed mice was disrupted, colonocytes and goblet cells disappeared, long crypts loosened, submucosa was edematous, and a large number of neutrophils infiltrated into the mucosa and submucosa, accompanied by crypt abscesses. However, NADD administration protected colon tissues from damage induced by DSS. Colonocytes, goblet cells and long crypts increased, the submucosa became thinner, and neutrophil infiltration was less prominent. These protective effects of NADD became more obvious with increasing concentration. Conversely, PAS was used to identify goblet cells by staining secreted mucin glycoproteins. Mucin layers are considered protective factors against colitis (Chen et al., 2019). Colons from normal and NADD group mice showed mucin glycoprotein (Figure 2B). NADD increased mucin glycoproteins in a concentration-dependent manner. The grading of histological score was refer to references (Cooper et al., 1993; Kim S. E. et al., 2019). The pathohistological scores increased and the number of goblet cells lost significantly in the DSS only group than Normal and NADD only groups. However, the administration of NADD significantly decreased the pathohistological scores and increased the number of goblet cells in a concentration-dependent manner (Figures 2C,D). NADD thus protected against morphological changes induced by DSS exposure.

### NADD Downregulated mRNA and Protein Levels of Proinflammatory Factors

**FIGURE 3:**
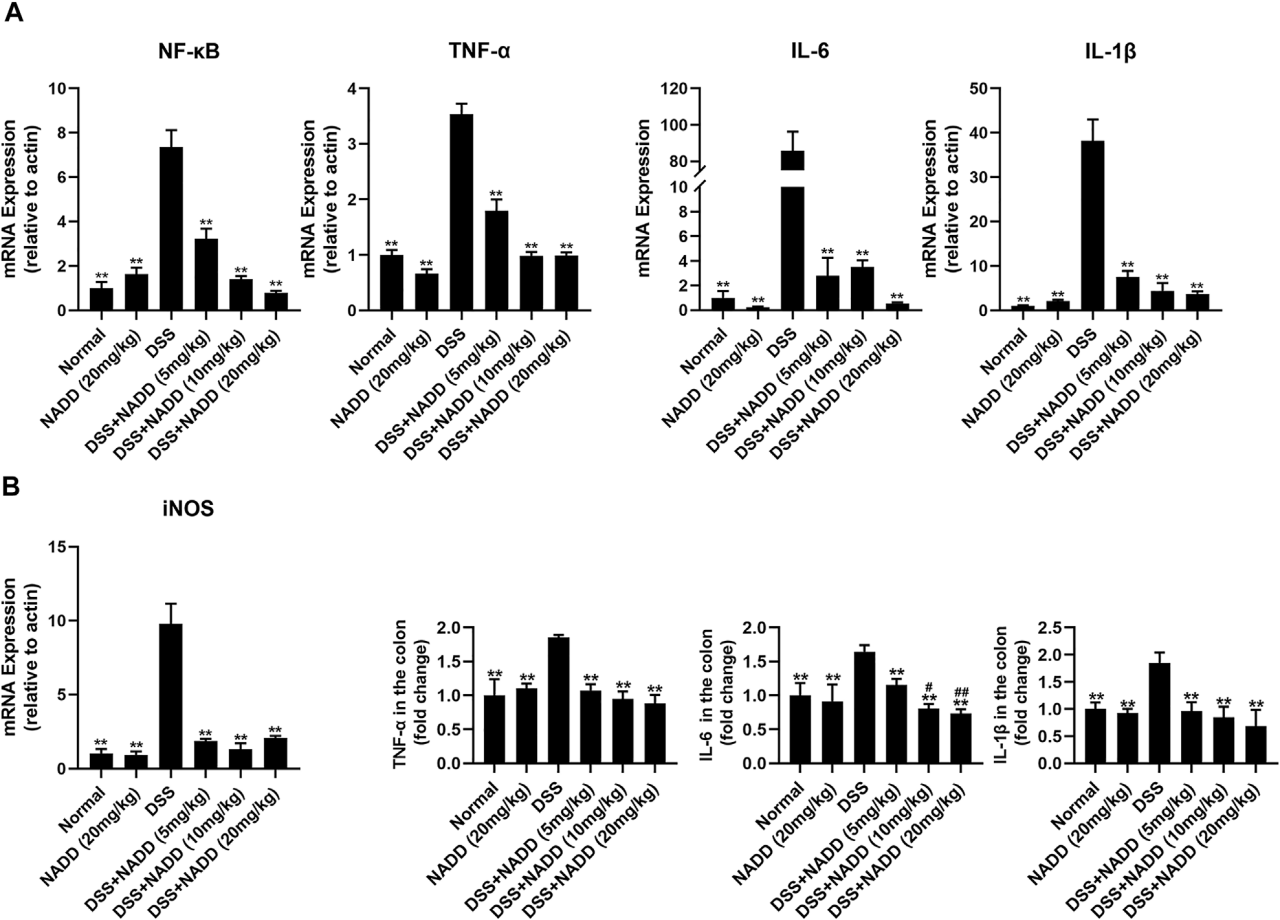
Gene expressions and protein levels of proinflammatory factors. **(A)** mRNA expression levels of NF-κB, TNF-α, IL-6, IL-1β, and iNOS in colon tissues from mice in different treatment groups. **(B)** ELISA results of proinflammatory factor levels, including TNF-α, IL-6, IL-1β, in colon tissues from mice in different treatment groups. Differences were statistically significant when compared with the DSS group mice (^*^ *p* < 0.05, ^**^ *p* < 0.01), and compared with the mice treated with DSS + NADD (5 mg/kg) (^*#*^ *p* < 0.01, ^*##*^ *p* < 0.01).

UC is typically accompanied by the secretion of various proinflammatory factors, including TNF-α, IL-6, IL-1β, and NF-κB. For example, NF-κB induces an increase in NO concentrations by activating inducible nitric oxide synthase (iNOS) (Kimura et al., 2000). In this study, transcription levels of NF-κB, TNF-α, IL-6, IL-1β and iNOS were increased significantly in the colon tissues of DSS group mice compared with that of the normal and NADD only group animals (^**^ *p* < 0.01). Thus, DSS exposure successfully induced inflammation of colon tissues in mice (Figure 3A). Transcription levels were downregulated in a concentration-dependent manner by NADD (^**^ *p* < 0.01). Similarly, protein levels of TNF-α, IL-6, and IL-1β were also highest in DSS group mice, and became lower following NADD treatment (^**^ *p* < 0.01). IL-6 levels of DSS + NADD (10 mg/ml) and DSS + NADD (20 mg/ml) groups also decreased significantly (^*#*^ *p* < 0.01, ^*##*^ *p* < 0.01) compared with mice treated with the low concentration of NADD (DSS + NADD (5 mg/ml)) (Figure 3B). NADD thus shows anti-inflammation activity for DSS-induced UC.

### NADD Inhibits LPS-Stimulated Inflammation in a Concentration-Dependent Manner

**FIGURE 4:**
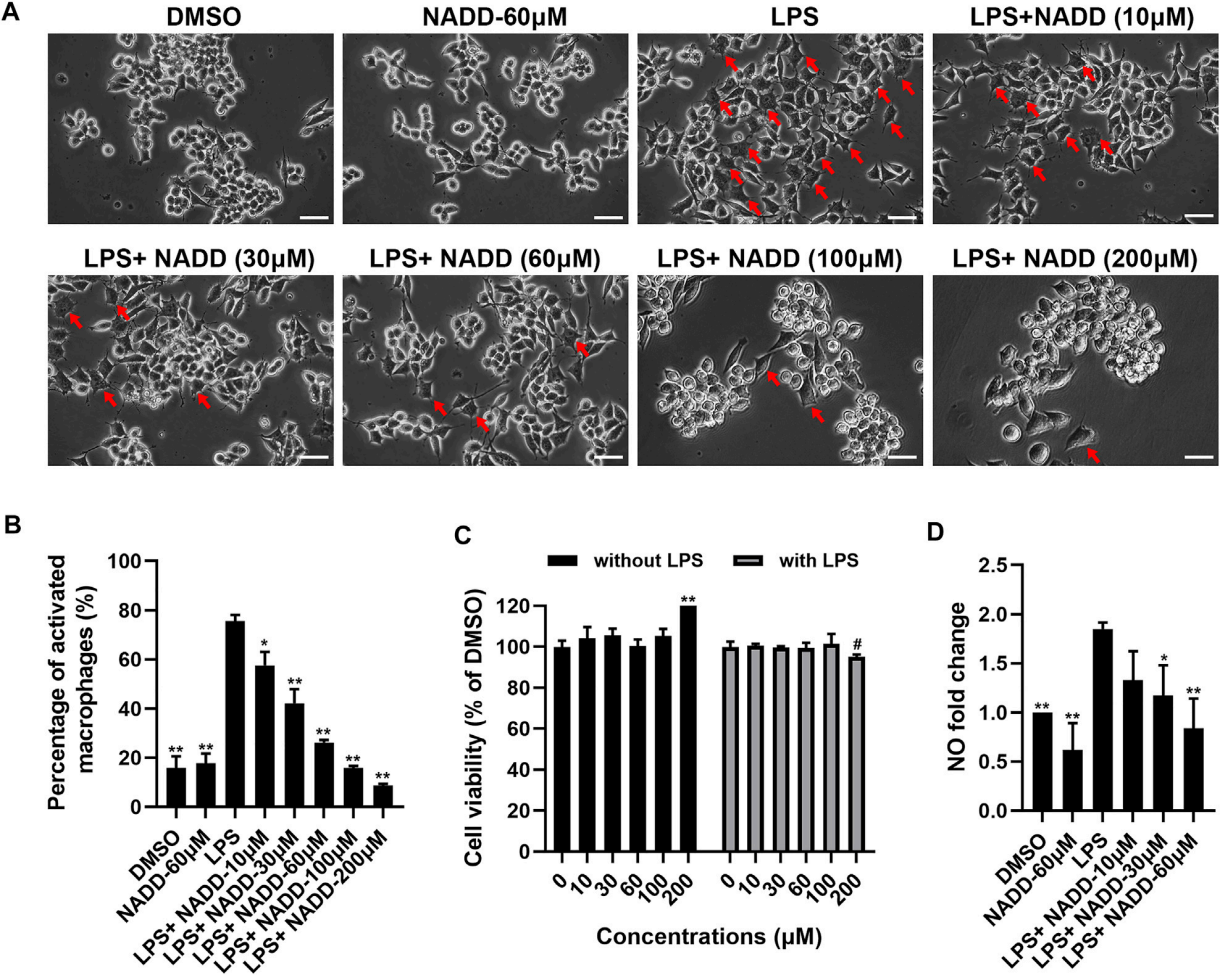
The effect of NADD on LPS-induced inflammation *in vitro*. **(A)** The morphology of RAW264.7 macrophages treated with DMSO, NADD-60 μM, LPS, LPS + NADD-10 μM, LPS + NADD-30 μM, LPS + NADD-60 μM, LPS + NADD-100 μM, and LPS + NADD-200 μM. Red arrows indicate activated macrophages; white bar = 100 μm. **(B)** The statistical analysis of morphologic changes of RAW264.7 macrophages treated with DMSO, NADD-60 μM, LPS, LPS + NADD-10 μM, LPS + NADD-30 μM, LPS + NADD-60 μM, LPS + NADD-100 μM, and LPS + NADD-200 μM. Differences were statistically significant when compared with the LPS group (^*^ *p* < 0.05, ^**^ *p* < 0.01). **(C)** Cell viability by CCK8; without LPS: RAW264.7 macrophages were not stimulated by LPS; with LPS: RAW264.7 macrophages were stimulated with LPS after cells were pre-treated with different concentrations of NADD. Differences were statistically significant when compared with the 0 μM group (^**^ *p* < 0.01, without LPS; ^*#*^ *p* < 0.05, with LPS). **(D)** NO in culture supernatant from different groups of cells. Differences were statistically significant when compared with the LPS group (^*^ *p* < 0.05, ^**^ *p* < 0.01).

RAW264.7 macrophages are a monocyte/macrophage-like cell linage (Kong et al., 2019) and LPS stimulates RAW264.7 to induce inflammation. This activity is accompanied by the expression of proinflammatory cytokines and iNOS (Plociennikowska et al., 2015; Kong et al., 2019). RAW264.7 macrophages activated by LPS change morphology from round to irregular and pseudopodia are expanded (Figure 4A). Cells treated with DMSO and 60 μM NADD were round and small, basically with no pseudopodia. After LPS exposure, most cells became irregular, cell bodies enlarged, and pseudopodia expanded. RAW264.7 macrophages treated with NADD showed reduced numbers of activated cells, and greater numbers of normal cells in a concentration-dependent manner. The statistical analysis showed that the morphological changes were significant as compared with the LPS group (Figure 4B). Further, NADD significantly decreased the generation of NO. This effect was also concentration-dependent (Figure 4D, ^*^ *p* < 0.05, ^**^ *p* < 0.01). Compared with 10 μM and 30 μM NADD, the higher concentration of NADD (60 μM) induced a better anti-inflammation (Figure 4D) effect without adverse impacts on cell phenotype and viability with or without LPS stimulation (Figures 4A,C). NADD thus shows anti-inflammatory activity *in vitro*.

### NADD Treatment Reversed LPS-Induced Changes of Gene Expressions

**FIGURE 5:**
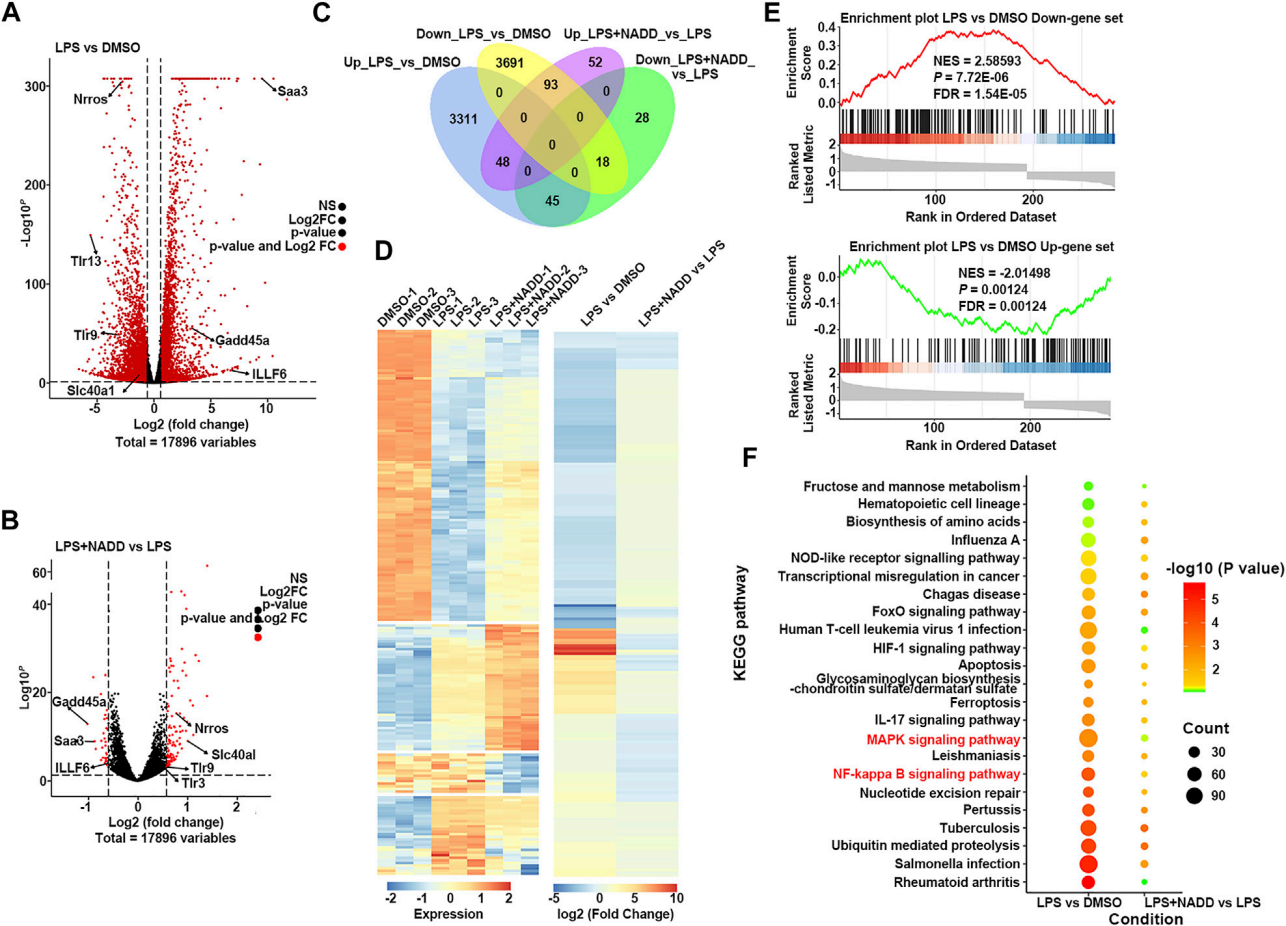
The result of RNA-seq. **(A)** The volcano plot of DEGs between LPS and DMSO treated RAW264.7 macrophages; The red dots show significantly DEGs. **(B)** Volcano plot of DEGs between LPS + NADD LPS treated RAW264.7 macrophages; the red dots show significantly DEGs **(C)** Venn diagrams of overlapping genes with LPS only treatment and LPS + NADD treated RAW264.7 macrophages. **(D)** Heatmap of 204 common DEGs in LPS and LPS + NADD treated RAW264.7 macrophages. **(E)** GSEA analysis result of DEGs. **(F)** KEGG enrichment analysis of DEGs. The DEGs were defined by |foldchange| > 2 and *p* < 0.05.

Transcriptome analysis was used to assess anti-inflammatory mechanisms of NADD. First, RAW264.7 macrophages were treated with LPS for 24 h to induce inflammation *in vitro*. Cells treated with DMSO served as a parallel control. Another group of cells were pre-treated with NADD (60 μM) for 1 h followed by LPS treatment for the next 24 h. Genome-wide profiles of differentially expressed genes (DEGs) were obtained by RNA-seq from all groups of cells. Cut-off criteria for volcano plots was fold change≥1.5 and *p* < 0.05 to identify DEGs. Many more DEGs were observed when comparing LPS and DMSO treated cells than when comparing LPS + NADD and LPS treated cells. Almost all genes downregulated by LPS treatment were upregulated after LPS treatment plus NADD treatment (Figures 5A,B), for example, *Nrros*, which downregulates ROS production (Bonini and Malik, 2014). Also, almost all genes upregulated by LPS treatment were downregulated after LPS treatment plus NADD treatment (Figures 5A,B), for example, *Saa3*, which is an inflammatory ligand that stimulates the production of IL-6 and TNF-α (Zhuang et al., 2017). The Venn diagrams (Figure 5C) showed 7,206 DEGs between LPS and DMSO treated cells, and 284 DEGs between LPS + NADD and LPS treated cells. Notably, almost 72% of the LPS + NADD affected genes (204/284) were also DEGs affected by LPS treatment alone. 93 DEGs that were down-regulated by LPS could be up-regulated by treatment of LPS + NADD, and 45 DEGs that were up-regulated by LPS could be down-regulated by treatment of LPS + NADD. The heat-map (Figure 5D) also shows that expression changes after LPS treatment are reversed by NADD treatment (fold change≥1.5, *p* < 0.05): Downregulated genes after LPS treatment were upregulated by NADD and vice-versa. Furthermore, gene set enrichment analysis (GSEA) illustrated the gene sets of up-regulated and down-regulated genes following LPS + NADD treatment (Figure 5E). As expected, NADD could reprogram the expressions of LPS regulated gene sets (Figure 5E). The intersection pathways enriched by both conditions were showed in Figure 5F, and the results showed that DEGs under both conditions were enriched in NF-kB and MAPK pathways. These findings suggest that NADD reverses LPS-induced inflammation at a transcriptional level. NADD inhibited LPS-induced Inflammation through TLR4/NF-kB and MAPK pathways.

**FIGURE 6:**
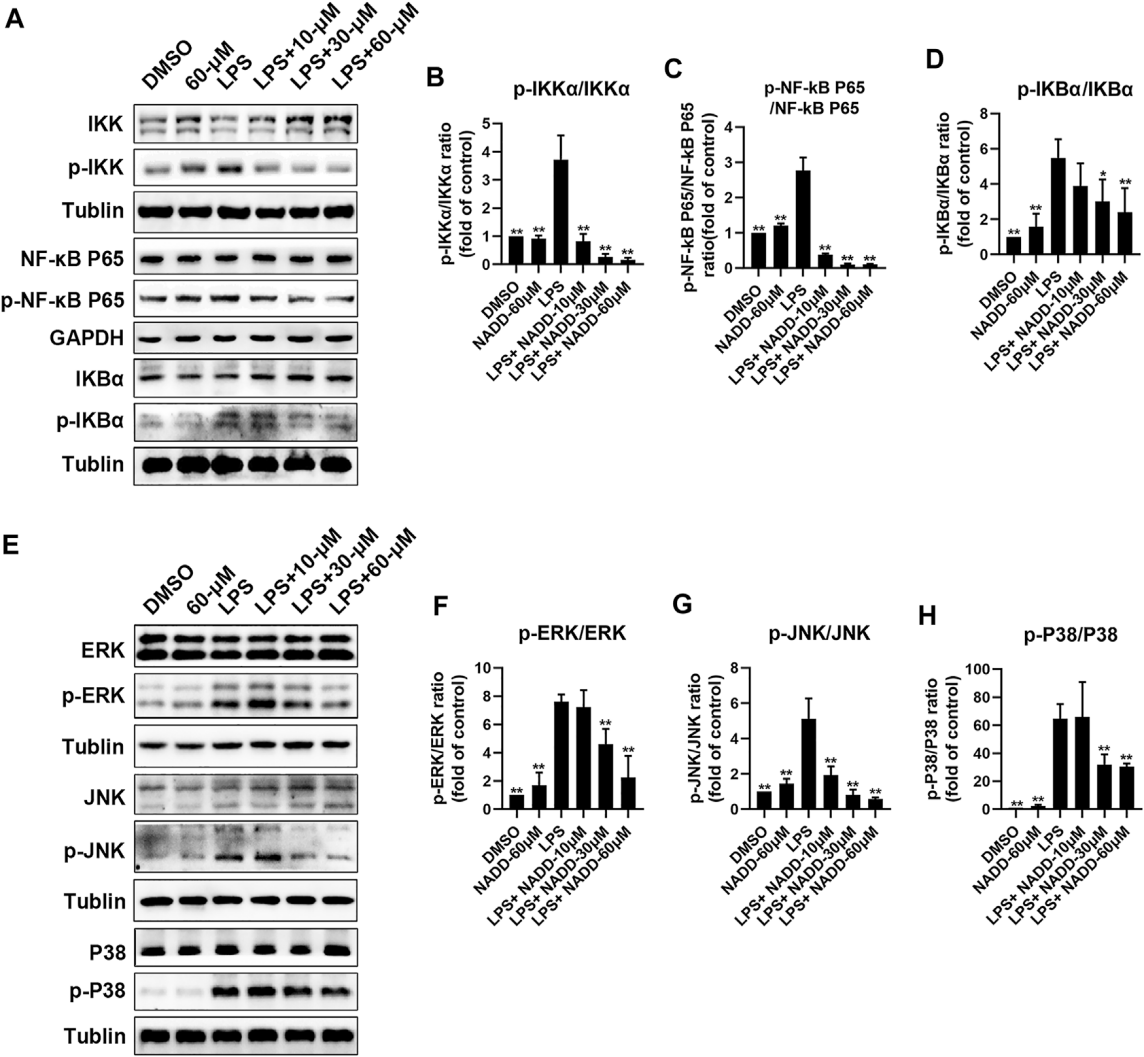
Protein levels of NF-kB and MAPK pathway factors after LPS and NADD treatments. **(A)** Expression of IKK, p-IKK (LPS stimulated 30 min), NF-kB p65, p-NF-kB p65 (LPS stimulated 60 min), IKBα and p-IKBα (LPS stimulated 60 min) in the NF-kB pathway. Tubulin were internal reference proteins. Bar charts showing expression of p-IKK/IKK **(B)**, p-NF-kB p65/NF-kB p65 **(C)** and p-IKBα/ IKBα **(D)**. **(E)** expressions of ERK, p-ERK (LPS stimulated 15 min), JNK, p-JNK (LPS stimulated 30 min), P38 and p-P38 (LPS stimulated 30 min) in the MAPK pathway; Tubulin was the internal reference protein; Bar charts showing expression of p-ERK/ERK **(F)**, p-JNK/JNK **(G)** and p-IKBα/IKBα **(H)**. Differences were statistically significant when compared with the LPS treated cells (^*^ *p* < 0.05, ^**^ *p* < 0.01).

RAW264.7 macrophages were treated with LPS and different concentrations of NADD to further explore anti-inflammatory effects of NADD *in vitro*. Western blotting was used to detect representative proteins involved in inflammatory signaling. Protein levels of p-IKK/IKK, p-NF-κB p65/ NF-κB p65, and p-IκBα/ IκBα in the NF-κB pathway were significantly elevated in LPS treated cells compared with DMSO and 60-μM treated cells. However, this effect was reversed significantly by NADD in a concentration-dependent manner (^*^ *p* < 0.05, ^**^ *p* < 0.01) (Figures 6A–D). LPS treatment also significantly upregulated protein levels of p-JNK/JNK, p-ERK/ERK and p-P38/P38 compared with DMSO and 60 μM treated cells, and NADD reduced the levels of these proteins in a concentration-dependent manner (^*^ *p* < 0.05, ^**^ *p* < 0.01) (Figures 6E–H). NADD thus suppress inflammation through modulation of NF-κB and MAPK pathways.

## Discussion

The etiology of UC is murky and complicated (Eisenstein, 2018). Hence, an effective strategy for screening compounds for useful clinical activity is to use pathological phenotype, including body weight, DAI, colon length, and histological changes. The latter may include disruption of epithelial barriers, goblet cell depletion, and damage to mucosa (Perse and Cerar, 2012; Chen et al., 2019). Damage to the epithelial barrier caused by UC allows gut microorganism invasion and enhances serious inflammation (Eisenstein, 2018). Hence, anti-inflammatory drugs are expected to be effective in treating UC. In this study, NADD was extracted from *I. cicadae*. The utility of this compound for treating UC has been observed and anti-inflammatory mechanisms were partially revealed.

NADD was found to significantly reduce DSS-induced DAI, improve colon length shortening, and reduce body weight loss. Especially, NADD decreases DAI, which reflects the main signs UC. Further, NADD administration significantly restored DSS-induced disruption or loss of the mucosal layer, columnar colonocytes, goblet cells and long crypts, and rescued submucosa edema and neutrophil infiltration into mucosa and submucosa. The actions of NADD were concentration-dependent. Therefore, NADD displayed the ability to relieve signs and symptoms of DSS-induced UC. In Figure 1F, although there were decreases of body weights in NADD group (Supplementary Figure S3), considered that NADD did not change the DAI when compared with normal group, we suspected that NADD may have a role of weight loss. However, the specific reason and underlying mechanism of decreasing of body weight need further studies in future.

DSS-induced UC is usually accompanied by disruption of the intestinal mucosal barrier accompanied by immune cell infiltration of mucosa and submucosa (Perse and Cerar, 2012). Further, bacterial invasion is also typical (Johansson et al., 2010). The main structural component of gram-negative bacteria is formed by LPS. The LPS receptor is the TLR4/MD-2 complex. This complex plays an important role in inflammation initiated by bacterial invasion (Kogut et al., 2005; St Paul et al., 2012; Yao et al., 2019). NF-κB is a downstream molecule of TLR4 signaling (Luo et al., 2012) and is often upregulated or over-activated in inflammatory disorders (Atreya et al., 2008; Danese and Mantovani, 2010). Activated NF-κB enters the nucleus and promotes the transcription of important cytokines (Thompson and Van Eldik, 2009; Cao et al., 2018). NF-κB is activated by phosphorylation, following ubiquitination and degradation of IκBs, IκBs phosphorylated by phosphorylated IKK (Atreya et al., 2008; Napetschnig and Wu, 2013). The MAPK pathway involves well-known proteins, such as P38, ERK, and JNK (Chang and Karin, 2001), TLR4 activation also activates this pathway (Hofmann et al., 2017). Briefly, TLR4 actives MyD88 and MyD88 activates TAK1. Subsequently, P38, ERK, and JNK are activated by TAK1, which activates activator protein 1 (AP-1). This protein promotes the production of proinflammatory cytokines and the initiation of inflammation (Palsson-McDermott and O'Neill, 2004; Moresco et al., 2011; Zhang et al., 2020).

In this study, NADD suppressed transcriptional and protein expression of proinflammatory cytokines, including TNF-α, IL-6 and IL-1β, and NF-κB induced by DSS *in vivo* (Figure 4). Although the pinnacle of LPS-induced transcriptional changes in macrophages is 4–6 h, we detected some interested gene changes of LPS^+/−^NADD on macrophages after 24 h, because we analyzed transcripts of TNF-α, IL-6 and IL-1β after 4 h of LPS^+/−^NADD treatment, the results showed that NADD reversed the increasing transcription level of TNF-α, IL-6 and IL-1β after 4 h of LPS treatment in a concentration dependent manner (Supplementary Figure S4). This result was consistent with the RNA-seq result. RNA-seq of macrophages showed that NADD reversed the gene signature generated by LPS exposure *in vitro*. Thus, results *in vivo* and *in vitro* are consistent. Subsequent western blotting showed that NADD significantly reduced phosphorylation of IKK, NF-κB p65, and IKBα in the NF-κB pathway, and suppressed the activation of JNK/ERK/P38 in the MAPK pathway. Considering the important role of LPS receptor TLR4 in the inflammation both *in vitro* and *in vivo*, we have predicted whether there was an interaction between NADD and TLR4 by molecular docking, and the result showed that NADD could docking with the pocket of the TLR4/MD-2 (Supplementary Figure S5), and this result indicated NADD may combine directly with the TLR4/M2 complexes, prevent them from combining to TLR4 receptor. Thus, TLR4 signaling may be prevented by NADD, as well as the downstream pathways NF-κB and MAPK. NF-κB and MAPK pathways are closely linked and therefore NADD may alleviate DSS-induced UC through modulation of both pathways.

## Conclusion

The present study shows that NADD significantly alleviates DSS-induced UC symptoms, such as increased DAI and histopathological changes including weight loss, colon length shortening, colonic tissue damage, as well as expression of proinflammatory factors *in vivo*. NADD also reverses LPS-induced gene signatures and LPS-related inflammatory signaling pathways including NF-κB and MAPK pathways *in vitro*. Hence, NADD may be a hit compound for UC therapy.

## Data Availability

The datasets presented in this study can be found in online repositories. The names of the repository/repositories and accession number(s) can be found below: https://www.ncbi.nlm.nih.gov, accession ID: PRJNA788753.
