# Supplementary material for: N-Acetyldopamine Dimer Attenuates DSS-Induced Ulcerative Colitis by Suppressing NF-κB and MAPK Pathways
Source: Front Pharmacol. 2022 Apr 6;13:842730. doi: 10.3389/fphar.2022.842730 (PMC9030057; doi:10.3389/fphar.2022.842730)
Supplement: Supplementary file 1 [file DataSheet1.PDF]

Supplementary Material

N-acetyldopamine Dimer Attenuates DSS-Induced Ulcerative Colitis by Suppressing NF-κB and MAPK Pathways

Li-jun Huang[1 †], Yu-mei Wang[1 †], Lei-qiang Gong[2], Chao Hu[1], Yu Gui[1], Chen Zhang[2], Xue Tan[1], Xian-kuo Yu[1], Yi-le Liao[1], Yan Luo[1], Yu-qin Tang[1], Yi-fei Dai[3], Yun Deng[2 *], Dong Wang[1 *] and Da-le Guo [2 *]

[1] State Key Laboratory of Southwestern Chinese Medicine Resources, School of Basic Medical Sciences, Chengdu University of Traditional Chinese Medicine, Chengdu, People's Republic of China

[2] School of Pharmacy, Chengdu University of Traditional Chinese Medicine, Chengdu, People's Republic of China Country

[3] Department of Basic Medical Sciences, School of Medicine, Tsinghua University, Beijing, People's Republic of China

† Co-first author: Li-jun Huang and Yu-mei Wang are contributed equally

*** Correspondence:**
Corresponding Author
dengyun@cdutcm.edu.cn (Yun Deng); dwang@cdutcm.edu.cn (Dong Wang);
guodale@cdutcm.edu.cn (Da-Le Guo)

1 Supplementary Data

1.1 sequence read archive (SRA) accession numbers of RNA-seq

Group	Accession number	Group	Accession number	Group	Accession number
DMSO_1	SAMN24021655	LPS_1	SAMN24021658	LPS+NADD_1	SAMN24021661
DMSO_2	SAMN24021656	LPS_2	SAMN24021659	LPS+NADD_2	SAMN24021662
DMSO_3	SAMN24021657	LPS_3	SAMN24021660	LPS+NADD_3	SAMN24021663

2 Supplementary Figures

2.1 The ^{1}H-NMR spectrum of NADD

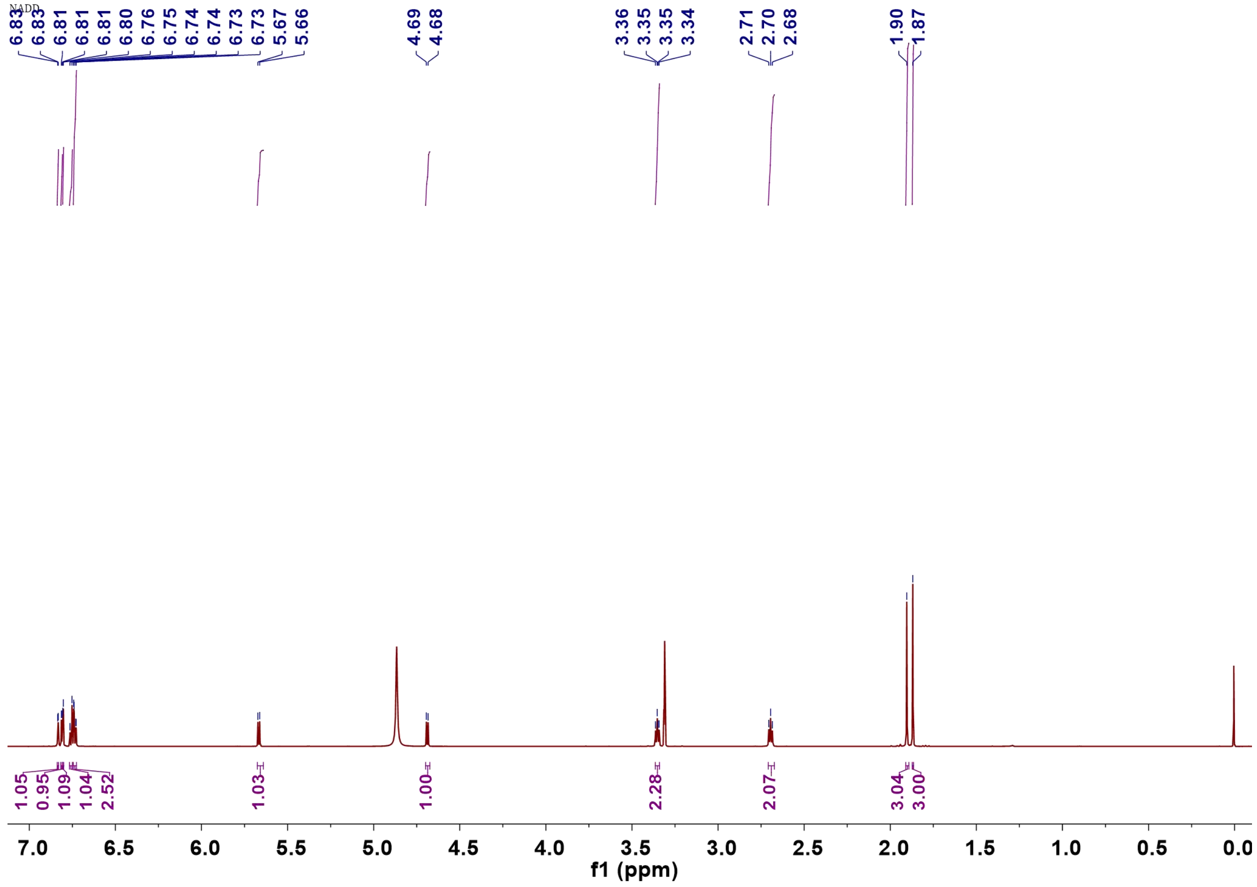

Supplementary Figure 1. ^{1}H-NMR spectrum (700 MHz) of NADD measured in CD_3OD with a Bruker 700 Ascend NMR instrument.

2.2 The ^{13}C-NMR spectrum of NADD

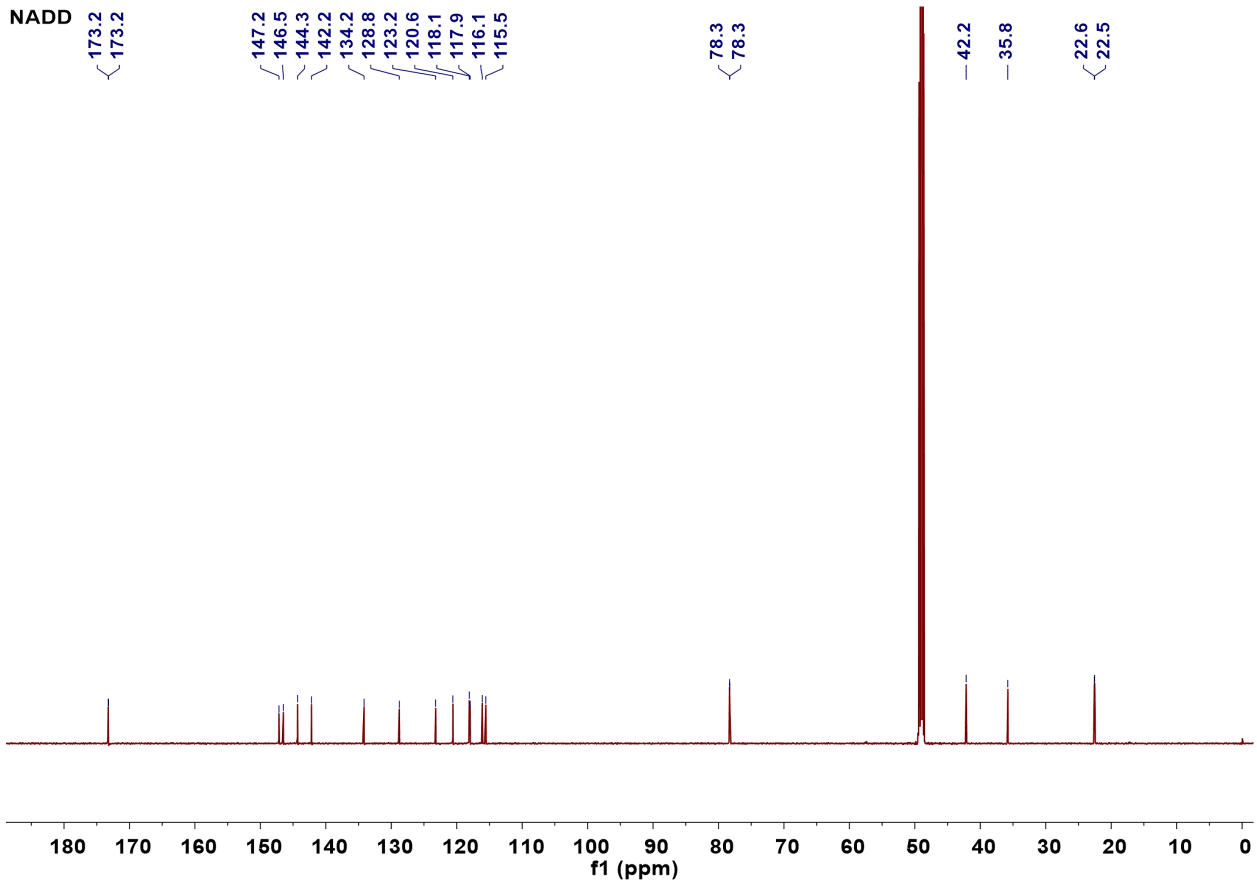

Supplementary Figure 2. ^{13}C-NMR spectrum (175 MHz) of NADD measured in CD_3OD with a Bruker 700 Ascend NMR instrument.

Supplymentary materials

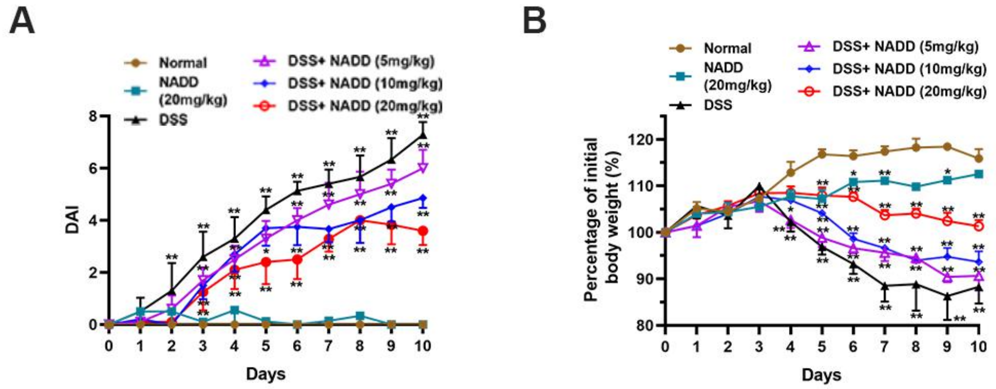

Supplementary Figure 3. DAI and body weight of mice among different treatment groups. **(A)** DAI of mice among different treatment groups. **(B)** Body weight changes of mice among different treatment groups. Differences were statistically significant when compared with mice in the Normal group ($^{*}P < 0.05$, $^{**}P < 0.01$).

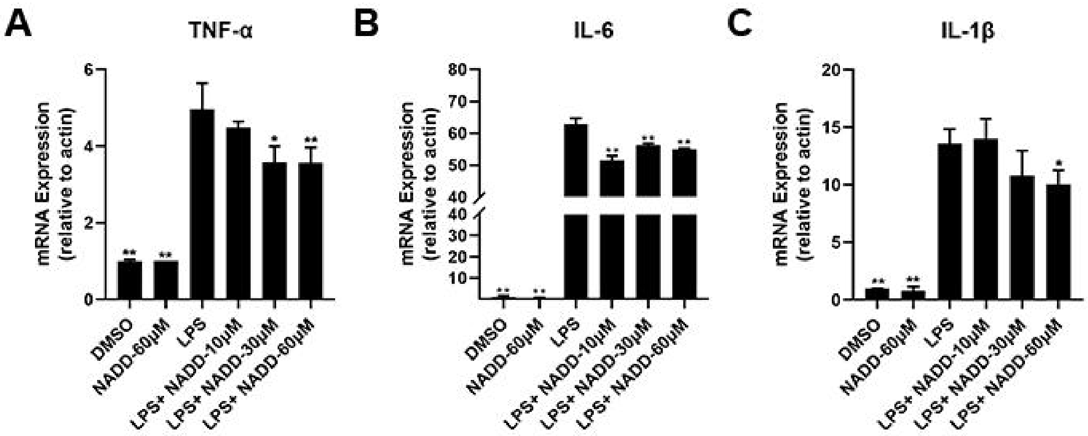

Supplementary Figure 4. The transcription level of TNF-α, IL-6 and IL-1β after 4 hours of LPS+/-NADD treatment of RAW 264.7 cells. **(A)** mRNA expression level of TNF-α in different treatment groups. **(B)** mRNA expression level of IL-6 in different treatment groups. **(C)** mRNA expression level of IL-1β in different treatment groups.

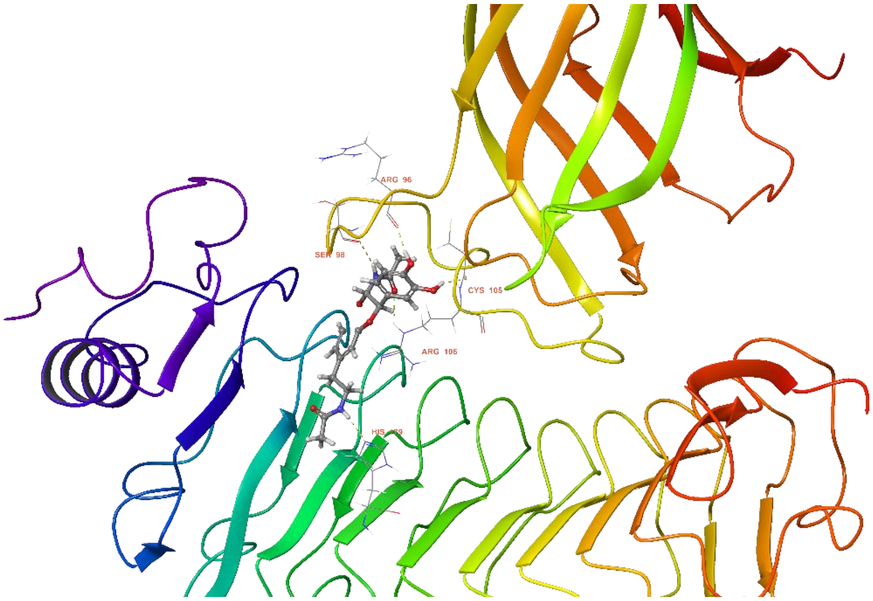

Supplementary Figure 5. Molecular docking results of NADD with TLR4/MD2.
